# Enhancing vs. inhibiting semantic performance with transcranial magnetic stimulation over the anterior temporal lobe: Frequency- and task-specific effects

**DOI:** 10.1016/j.neuroimage.2021.117959

**Published:** 2021-07-01

**Authors:** JeYoung Jung, Matthew A. Lambon Ralph

**Affiliations:** a School of Psychology, University of Nottingham, University Park, Nottingham NG7 2RD, UK; b MRC Cognition and Brain Science Unit (CBU), University of Cambridge, Cambridge CB2 7EF, UK

**Keywords:** Anterior temporal lobe, Repetitive transcranial magnetic stimulation, Semantic representation, Semantic enhancement, Neuroplasticity

## Abstract

Accumulating, converging evidence indicates that the anterior temporal lobe (ATL) appears to be the transmodal hub for semantic representation. A series of repetitive transcranial magnetic stimulation (rTMS) investigations utilizing the ‘virtual lesion’ approach have established the brain-behavioural relationship between the ATL and semantic processing by demonstrating that inhibitory rTMS over the ATL induced impairments in semantic performance in healthy individuals. However, a growing body of rTMS studies suggest that rTMS might also be a tool for cognitive enhancement and rehabilitation, though there has been no previous exploration in semantic cognition. Here, we explored a potential role of rTMS in enhancing and inhibiting semantic performance with contrastive rTMS protocols (1 Hz vs. 20 Hz) by controlling practice effects. Twenty-one healthy participants were recruited and performed an object category judgement task and a pattern matching task serving as a control task before and after the stimulation over the ATL (1 Hz, 20 Hz, and sham). A task familiarization procedure was performed prior to the experiment in order to establish a ‘stable baseline’ prior to stimulation and thus minimize practice effect. Our results demonstrated that it is possible to modulate semantic performance positively or negatively depending on the ATL stimulation frequency: 20 Hz rTMS was optimal for facilitating cortical processing (faster RT in a semantic task) contrasting with diminished semantic performance after 1 Hz rTMS. In addition to cementing the importance of the ATL to semantic representation, our findings suggest that 20 Hz rTMS leads to semantic enhancement in healthy individuals and potentially could be used for patients with semantic impairments as a therapeutic tool.

## 1 Introduction

Concepts and meaning are fundamental components of human cognition. We use this knowledge every day to recognise objects in our environment, to anticipate how they will behave and interact with each other and, use them to perform functions, to generate expectations for situations, and to interpret language. Converging evidence from neuropsychological and neuroscientific studies indicates that the anterior temporal lobe (ATL) is a central area serving as a representational hub interacting with distributed modality-specific ‘spoke’ regions in order to form coherent and generalizable concepts (for a review, see Lambon Ralph, 2014; Lambon Ralph et al., 2010; Patterson et al., 2007; Ralph et al., 2017). Semantic dementia (SD; the temporal lobe variant of frontotemporal dementia) is a most striking example supporting this hypothesis – SD patients with atrophy centred in the ATL exhibit a selective semantic impairment in both verbal and non-verbal domains (Bozeat et al., 2000; Hodges and Patterson, 2007; Mummery et al., 2000). This hypothesis was subsequently supported and extended by recent investigations using intracranial recording (Abel et al., 2015; Chen et al., 2016; Shimotake et al., 2015), magnetoencephalography (MEG) (Clarke et al., 2011; Mollo et al., 2017), and functional magnetic resonance imaging (fMRI) (Coutanche and Thompson-Schill, 2015; Murphy et al., 2017; Peelen and Caramazza, 2012; Visser et al., 2010).

A crucial form of convergent evidence for the causal role of the ATL in semantic representation came through a series of experiments with healthy participants using repetitive transcranial magnetic stimulation (rTMS) demonstrating that rTMS over the ATL causes transient impairments in various semantic tasks (Binney et al., 2010; Jackson et al., 2015; Jung and Lambon Ralph, 2016; Lambon Ralph et al., 2009; Pobric et al., 2010a, 2007, 2010b; Pobric et al., 2009). Although this ‘virtual lesion’ rTMS approach has been useful to verify brain-behaviour relationships, several studies have shown enhancement in cognitive performance, suggesting that rTMS is also capable of facilitating cortical activity at the site of simulation (for a review, see Vallar and Bolognini, 2011), depending on the type and frequency of stimulation (Miniussi and Rossini, 2011). Furthermore, there is a growing interest in the possibility of utilising TMS in cognitive rehabilitation (Miniussi and Rossini, 2011; Rossi and Rossini, 2004) and enhancement (Luber and Lisanby, 2014). In this study, therefore, we tested various rTMS protocols for enhancing vs diminishing semantic processing.

Converging evidence indicates that rTMS below 1 Hz reduces cortical excitability at the target region (Chen et al., 1997), whereas high frequency (HF) rTMS (typically between 5 and 20 Hz) can increase it (Pascual-Leone et al., 1994). With this ability, rTMS has been widely used to manipulate cortical processing and to examine the resultant changes in cognitive performance. Studies reported TMS-induced performance enhancements have employed various TMS protocols including single pulse, theta burst, paired pulse, and rTMS at both low and high frequencies and cognitive tasks (for a review, see Luber and Lisanby, 2014; Miniussi and Rossini, 2011). Cortical processing are affected differently by these various forms of TMS: some disrupting processing through the addition of neural noise; briefly inhibiting or facilitating activity; whilst others modulate cortical excitability up or down for periods beyond the stimulation. Luber and Lisanby (2014) suggested that there are three mechanisms underlying TMS enhancement effects: nonspecific effects of TMS, disruption of competing processing (i.e., addition-by-subtraction), and direct modulation of TMS to task-related cortex. Non-specific TMS effects are ‘side effects’ of the stimulation (e.g., increased alertness following the ‘click’ sound or tactile sensation). These peripheral sensations can cause intersensory facilitation that contributes to performance enhancements (Terao et al., 1997). An example of ‘addition-by-subtraction’ is that inhibitory 10mins 1 Hz rTMS applied to the right posterior parietal cortex (involved in directing attention to salient stimuli) improved reaction time (RT) in a visual search task when there are attention-capturing distracters (Hodsoll et al., 2009). Many HF rTMS studies (between 5 and 20 Hz and iTBS; intermittent theta burst stimulation) have successfully produced performance enhancements via direct TMS modulation to task-related cortex (Ahn et al., 2013; Boyd and Linsdell, 2009; Hoy et al., 2016; Hwang et al., 2010; Ragert et al., 2003; Wagner et al., 2006). The associated, prolonged facilitatory effects are thought to be based on long term potentiation (LTP) (Bliss and Lomo, 1973). Here, we attempted to achieve semantic performance enhancements via direct modulation by stimulating the ATL with HF rTMS, and contrasted this to the transient inhibition induced by low-frequency stimulation to the same region, in the same participants.

We also tackled a crucial, additional cognitive factor that is rarely addressed in rTMS enhancements studies, namely practice effects (performance enhanced through repeated exposure to the test procedure and stimuli). Many rTMS enhancements studies have sought to measure changes in behavioural performance by comparing two sessions, before and after stimulation. However, the effects of practice at such brief test-retest intervals have not been considered as a significant factor in TMS literature. Practice effects on cognitive performance vary according to the difficulty of the task (stronger for more difficult tasks), the length of the test-retest interval (practice effects diminish over time), the individual's ability at the time of testing (stronger practice effects for weaker participants) and can be reduced through the use of alternative forms or stimuli sets (Basso et al., 1999; Benedict and Zgaljardic, 1998; Dikmen et al., 1999) though practice-related performance improvement can still persist (Kay, 1991). Studies investigating practice effects on cognitive test performance suggest that at least two assessments need to be conducted before performance stabilizes and there are no practice effects on simple tasks with longer (i.e., one week) test-retest intervals (Collie et al., 2003; Falleti et al., 2006). Accordingly in the current study, we asked participants to perform a thorough task familiarization procedure in order to establish a ‘stable baseline’ prior to stimulation and thus minimize practice-related improvement in the experiment itself (see Materials and Methods).

Given that there is no consensus on a set rTMS protocol for inducing facilitatory effects, we first evaluated various rTMS protocols that have produced performance enhancements on higher cognition functions in healthy participants. The four selected protocols (5, 10, and 20 Hz, and iTBS) have showed facilitatory effects on working memory, learning and executive functions in previous investigations (see the Materials and Methods). In the pilot study, we applied rTMS with four different HF over the left ventrolateral ATL (vATL) in healthy participants to identify which protocols induce semantic performance enhancements. Early rTMS studies stimulated at the lateral ATL, 10 mm posterior to the tip of the temporal pole on the middle temporal gyrus on the basis that this fell into the area of atrophy observed in SD patients (Lambon Ralph et al., 2009; Pobric et al., 2010a, 2007). Recent convergent evidence from patient, fMRI and cortical electrode has shown that the vATL region appears to be the centre point of hub with strong multimodal and omni-category responses (Lambon Ralph et al., 2017; Rice et al., 2015). Also, our recent rTMS-fMRI combined study demonstrated that stimulating the vATL decreased regional activity at the target site and the ventromedial ATL, and induced slowed semantic performance (Jung and Lambon Ralph, 2016). Therefore, we chose the vATL as the target site in this study. Having selected 20 Hz rTMS from the pilot study, we investigated its effects on semantic performance in comparison to an opposing, inhibitory stimulation (1 Hz) and sham stimulation. As a within-subject design, before and after the stimulation, all participants performed an object category judgement task and a pattern matching task serving as a control task. We hypothesized that 20 Hz rTMS over the vATL would produce semantic performance enhancements (faster RT) in comparison to no-stimulation (before the stimulation) and sham stimulation, whilst 1 Hz rTMS would show the opposite effects on semantic performance. In addition, we expected that semantic processing should be preferentially modulated by both 20 Hz and 1 Hz rTMS over the left vATL.

## 2 Materials and methods

### 2.1 Participants

Twenty one healthy participants participated in this study (7 females, mean age, 22 ± 3.1 years). The sample size was calculated based on a previous study (Jung and Lambon Ralph, 2016), which used a 2 (TMS to ATL vs. control site) × 2 (semantic vs. control task) within subject design. These previous data (collected in *N* = 23 participants) indicated that to achieve α=0.05, power=80% for the critical interaction between TMS site and task then N≥18 were required. The observed effect size for the interaction was 0.36. All participants were native English speakers with right-handed assessed by the Edinburgh Inventory for Handedness (Oldfield, 1971). They received a detailed explanation of the study and gave written informed consent prior to the experiment. The experiment was approved by the local ethics committee.

### 2.2 Experimental design and procedure

**Fig. 1:**
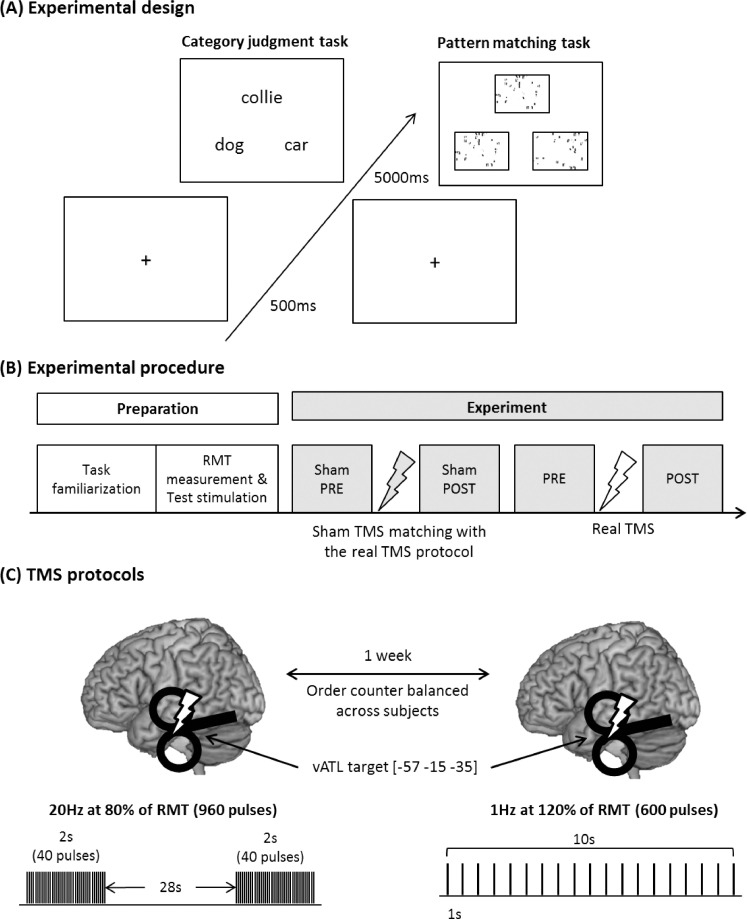
(A) Experimental design. Each trial starts with a fixation followed by stimuli, which have 3 items, a target on the top and 2 choices at the bottom. (B) Experimental procedure. Experimental preparation includes the task familiarization and RMT measurement/test stimulation for following the experiment. During the task familiarization, participants performed 120 trials of each task in order to saturate their task performance thereby minimizing practice effects. During the test stimulation, 100 pulses of TMS matched with the following TMS protocol were delivered over the occipital pole so as to prevent non-specific TMS effects. In the experiment, 4 task sessions were conducted before and after sham and real TMS stimulation. (C) TMS protocols. 2 TMS protocols were employed: 20 Hz for the facilitatory effects and 1 Hz for the inhibitory effects. Each protocol was delivered on different days with a week gap at least. The stimulation was applied at the ventrolateral anterior temporal lobe.

Participants performed an object category judgment task and a pattern matching task as a control task. The stimuli for the category judgment task were from the *Levels of Familiarity, Typicality, and Specificity (LOFTS)* semantic battery (Rogers et al., 2015). The 120 items probe semantic knowledge at the subordinate level and cover a variety of categories, including animals, vehicles, tools, foods, and plants. Participants were asked to indicate which of two categories was appropriate for a target object (e.g., target: collie, choice 1: dog, choice 2: car). In each trial, three words were presented on the screen, a target on the top and 2 choices at the bottom (Fig. 1A). As a control task, we employed a pattern matching task from previous TMS and fMRI studies (Pobric et al., 2007; Visser et al., 2012) which provided a better match to the categorisation task in terms of general difficulty. In this pattern matching task, participants were asked to select which of two patterns was identical to a target pattern (Fig. 1A). In order to minimize the practice effects, participants performed a task familiarization procedure in which they completed 120 trials of each task prior to the experiment. Participants then performed the main tasks before and after sham and real TMS (Fig. 1B). The task included 60 trials in the main experiments. A trial started with 500 ms fixation and the stimuli were presented until the participant's response or a maximum of 5000 ms. E-prime software (Psychology Software Tools Inc., Pittsburgh, PA, USA) was used to display stimuli and to record responses. The experimental design and procedure is summarized in Fig. 1.

### 2.3 Anatomical MRI acquisition

A high-resolution T1-weighted anatomical image collected on a 3T Philips MR Achieva scanner was obtained for all participants to guide a target site. The structural image was acquired using a 3D MPRAGE pulse sequence with 200 slices, in planed resolution 0.94 × 0.94, slice thinkness 0.9 mm, TR = 8.4 ms, and TE = 3.9 ms.

### 2.4 Transcranial magnetic stimulation (TMS)

A MagStim Super Rapid stimulator (The MagStim Company, Whitland, UK) was used to deliver stimulation with a figure of eight coil (70 mm). Resting motor threshold (RMT) was defined as a minimal intensity of stimulation inducing twitches in the contralateral first dorsal interosseous muscle of the right hand in at least 5 of 10 stimulations at rest. The average RMT intensity was 60.7% ± 7.2 in the experiment.

The target site [MNI: Montreal Neurological Institute, −57 −15 −35] was selected from previous fMRI and TMS studies (Jung and Lambon Ralph, 2016; Visser et al., 2012). The coordinate was located on the ventrolateral ATL (Fig. 1C) and transformed to each participant's native space. Statistical Parametric Mapping software (SPM8, Wellcome Trust Centre for Neuroimaging, London, UK) was used to normalize participants’ MRI scan against the MNI template and to convert the target coordinate to the untransformed individual native space coordinate using the inverse of each resulting transformation. These native space coordinates guided the frameless stereotaxy, via a Brainsight TMS-MRI co-registration system (Rogue Research, Montreal, Canada).

### 2.5 TMS protocol

In order to determine an optimal rTMS protocol, we had a pilot study testing four protocols that have shown facilitatory effects in previous studies: 5 Hz, 10 Hz, 20 Hz, and iTBS. We selected these protocols on the basis that a single session application had induced facilitatory effects on cognitive behaviours or motor evoked potentials. 5 Hz protocol had 2 blocks of 9 trains of 10 s stimulation repeated every 20 s (total 900 pulses) (Sole-Padulles et al., 2006). 10 Hz protocol had 3 blocks of 15 trains of 2 s stimulation repeated every 12 s (total 900 pulses) (Ahn et al., 2013). 20 Hz stimulation consisted of 3 blocks of 8 trains of 2 s stimulation repeated every 28 s (total 960 pulses) (Wagner et al., 2006). iTBS had 3 pulses of stimulation given at 50 Hz, a 2 s train of TBS repeated every 10 s for 190 s (total 600 pulses) (Hoy et al., 2016; Huang et al., 2005). As a control, we used sham stimulation in which one wing of a figure-eight coil was in contact with the target site, but at a 90° tilt from tangential (Lisanby et al., 2001). All protocols were delivered with 80% of RMT for each individual. In the pilot study, participants took part in 5 TMS sessions on different days (3 days gap). The order of protocols was counterbalanced across the participants. We conducted paired *t*-test between four facilitatory protocols and sham. The results demonstrated that 20 Hz stimulation induced marginally significant facilitatory effects on semantic processing (*N* = 4, 3 females, mean age = 23 ± 3.5yrs, Wilcoxon signed-rank test: *Z* = −1.5, *p* = 0.07) (Fig. S1). Therefore, we selected 20 Hz protocol for this experiment.

In the current experiment, we contrasted 20 Hz and 1 Hz rTMS, and compared these to each other as well as sham to stimulation. 1 Hz rTMS to the ATL (total 600 pulses, 120% RMT) has demonstrated inhibitory effects in semantic processing (Pobric et al., 2010a, 2007, 2010b; Pobric et al., 2009). Here, we expected to replicate the same 1 Hz rTMS effect whilst finding the opposite effect for 20 Hz stimulation. Sham TMS was delivered with the same protocol of the real TMS on the day of experiment. During the stimulation, participants were asked to be relaxed with closed eyes. Each session was conducted at the same time on different days with a week gap between sessions (Fig. 1C). In order to control for non-specific TMS effects, we delivered 60 pulses of stimulation (test stimulation, matched with the subsequent real TMS protocol) over the occipital pole prior to the experiment, which was localised using the international 10–20 system. Occipital pole is a common control site for TMS studies and previous studies have demonstrate that this site successfully served as the control site and did not influence behavioural performance and neural changes in either semantic or control (visual) tasks (Jung and Lambon Ralph, 2016; Lambon Ralph et al., 2009; Pobric et al., 2007, 2010b). Participants were instructed that they would receive different protocols of TMS varying the frequency and intensity in this study. After the experiment, we asked participants whether they could distinguish between sham and real TMS. All participants reported that all stimulations were real regardless of sham or real TMS. There were no complaints about discomfort associated with HF rTMS over the vATL.

### 2.6 Statistical analysis

The impact of vATL rTMS on performance was investigated in terms of reaction time and accuracy using repeated measures analysis of variance (ANOVA) and planned paired t-tests for the comparison of vATL rTMS effect (i.e. 1 Hz vs. 20 Hz, 1 Hz vs. sham, 20 Hz vs. sham). Comparisons were made between pre and post session within a group. Practice effect was evaluated using one-way ANOVA and post-hoc paired t-tests.

## 3 Results

**Fig. 2:**
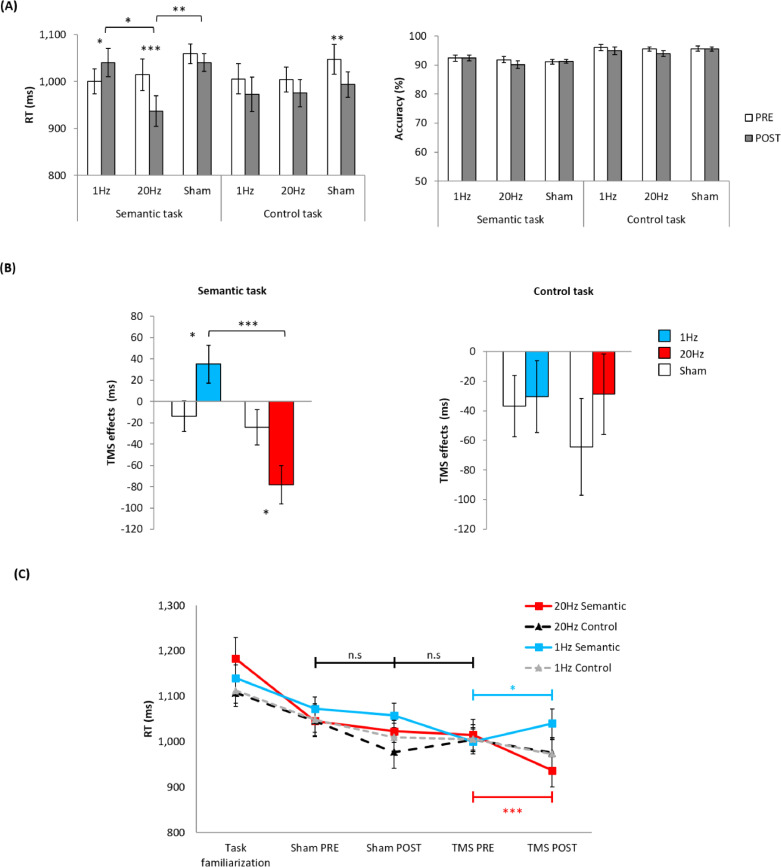
The results of the experiment. (A) The averaged reaction time (RT) and accuracy (%) for the semantic (category judgement) and control (pattern matching) tasks before (PRE) and after (POST) stimulation. White bars represent the PRE sessions and grey bars, the POST sessions. (B) TMS effects in the semantic and control task. TMS effects were computed by subtracting RT in PRE from POST TMS sessions. White bars represent the matched sham stimulation. Light blue coloured bars indicate 1 Hz stimulation and red bars, 20 Hz stimulation. (C) Practice effects. Squares with solid lines indicate semantic task performance, whereas triangles with dotted lines, control task performance. Error bar represents the standard error. * *p* < 0.05, *** *p* ≤ 0.001.

The participants’ performance on the semantic task (category judgement) and the control task (pattern matching) was compared following 20 Hz, 1 Hz, and sham vATL rTMS. Reaction time (RT) was examined using a repeated measures analysis of variance (ANOVA) with protocol (20 Hz, 1 Hz, vs. sham), task (category judgement vs. pattern matching), and TMS (Pre vs. Post) as within-subject factors. It should be noted that there were 2 sham stimulations (prior to 1 Hz and 20 Hz stimulation). As 2 sham stimulations did not differ in each PRE and POST sessions, we averaged them and used it as ‘sham stimulation’ in this analysis (Fig. S3). There were a significant main effect of protocol (F_2,__19_ = 15.41, *p* < 0.001, η^2^ = 0.62) and TMS (F_1,__20_ = 9.90, *p* = 0.005, η^2^ = 0.33) and interactions between the protocol and TMS (F_2,__19_ = 4.04, *p* = 0.035, η^2^ = 0.30) and between the protocol, task, and TMS (F_2,__19_ = 3.66, *p* = 0.045, η^2^ = 0.29). The other main effects and interactions did not reach the significance level (Fs < 2.37, ps > 0.14). Planned paired t-tests were performed between Pre and Post session as well as between the Post sessions (20 Hz, 1 Hz, and sham) to examine the interaction effect. We found that RT for the category judgements was slower after the 1 Hz stimulation (*t* = −2.40, *p* = 0.027, Cohen's *d* = 0.53) and faster after the 20 Hz stimulation (*t* = 4.37, *p* < 0.001, Cohen's *d* = 0.95) (Fig. 2A Left). The facilitatory effect after 20 Hz stimulation was significant compared to 1 Hz (*t* = 2.60, *p* = 0.017, Cohen's *d* = 0.56) and sham stimulation (*t* = −3.05, *p* = 0.006). In addition, RT for the pattern matching task was significantly faster after the sham stimulation (*t* = 3.24, *p* = 0.004, Cohen's *d* = 0.66) (Fig. 2A Left).

Accuracy rates were high (PRE-session: category task 92%, pattern matching 95%). ANOVA with protocol (20 Hz, 1 Hz, vs. sham), task (category judgement vs. pattern matching), and TMS (Pre vs. Post) as within-subject factors was performed on the accuracy rate. The results revealed a significant main effect of task (F_1,__20_ = 41.55, *p* < 0.001, η^2^ = 0.66) and TMS (F_1,__20_ = 5.00, *p* = 0.037, η^2^ = 0.20). Participants made less errors in the pattern matching than category judgment (Fig. 2A Right). However, there was no effect of protocol or an interaction (Fs < 1.42, ps > 0.27).

As noted in the Introduction, TMS-induced facilitatory effects need to be verified over and above practice effects. Accordingly, we expected that the facilitatory effect induced by TMS should be bigger than practice effect. To test this hypothesis, we calculated the TMS effects (RT difference between pre and post session: POST- PRE) and compared them with the RT changes caused by sham stimulation. The results are summarized in Fig. 2B. Planned paired t-tests were conducted between rTMS protocols and the matched sham stimulation. We found that the category judgment times were significantly slower after 1 Hz stimulation (*t* = 2.50, *p* = 0.021, Cohen's *d* = 0.54) and faster after 20 Hz stimulation (*t* = −2.21, *p* = 0.039, Cohen's *d* = 0.48) compared to the sham stimulation. There was a significant difference between 20 Hz and 1 Hz stimulation (*t* = 4.09, *p* = 0.001, Cohen's *d* = 0.89). As expected, there was no differences in the TMS effect for the pattern matching task (ps> 0.17) (Fig. 2B).

Sham stimulation performance was examined using ANOVA with protocol (1 Hz vs. 20 Hz), task (category judgement vs. pattern matching) and TMS (PRE vs. POST). In RT, there was a significant main effect of TMS (F_1,__20_ = 13.69, *p* = 0.002, η^2^ = 0.42), but no effect of other factors and interactions (Fs 〈 3.46, ps 〉 0.07). The effect of TMS reflects practice effects, i.e., a decrease in RT after any stimulation protocols regardless of tasks. In accuracy, there was a significant main effect of task (F_1,__20_ = 46.49, *p* < 0.001, η^2^ = 0.71), but no effect of other factors and interactions (Fs < 2.45, ps > 0.09). Again, the accuracy rates were high in both tasks (Pre-session: category judgement 91%, pattern matching 95%).

To evaluate practice effects between sessions (task familiarization, sham PRE and POST, and TMS PRE), we conducted a one-way ANOVA for each task. Fig. 2C summarises the results. In RT, we found practice effects (1 Hz semantic task F_3,__80_ = 2.88, *p* = 0.041, η^2^ = 0.33, 1 Hz control task: F_3,__80_ = 2.47, *p* = 0.068, η^2^ = 0.30, 20 Hz semantic task: F_3,__80_ = 5.36, *p* = 0.002, η^2^ = 0.44, 20 Hz control task: F_3,__80_ = 3.17, *p* = 0.029, η^2^ = 0.34). Post hoc paired t-tests demonstrated that there was no difference between the second and third sessions and between the third and fourth sessions. There was no significant practice effect in accuracy (Fs < 0.88, ps > 0.22).

## 4 Discussion

There is a growing interest in TMS as a tool for cognitive enhancement and rehabilitation, in addition to the more established use for evaluating the effect of TMS-induced cortical inhibition on cognitive function. Many studies employing various TMS paradigms have showed a facilitatory effect on motor and cognitive functions such as memory, language, executive functions (Luber and Lisanby, 2014; Rossi and Rossini, 2004). To date, however, TMS-induced enhancements have not been explored in the semantic domain. Depending on the TMS frequency, we found that semantic performance could be improved (20 Hz) or impeded (1 Hz) in the same participants. We also confirmed that the enhancing effect of 20 Hz rTMS over the vATL was not due to uncontrolled nuisance effects, including practice effects. Our results suggest that 20 Hz rTMS over the vATL is an optimal protocol for facilitating cortical processing during semantic categorisation and thus might be a beneficial intervention for semantic enhancement in healthy individuals and rehabilitation in patients.

To our best knowledge, this is the first study to demonstrate (a) opposing effects of vATL TMS on semantic performance in the same participants dependent on the frequency used; and (b) semantic enhancements in healthy participants by stimulating the vATL with a facilitatory protocol. The ability to both inhibit and improve semantic performance provides further strong evidence for the role of the ATL as a transmodal hub for semantic representation (Lambon Ralph, 2014; Rice et al., 2015). The demonstration of enhanced semantic performance is a crucial addition to the multiply replicated effect of inhibitory rTMS over the ATL (1 Hz rTMS and cTBS; continuous theta burst stimulation) during various semantic tasks (Jung and Lambon Ralph, 2016; Lambon Ralph et al., 2009; Pobric et al., 2010a, 2007, 2010b; Pobric et al., 2009). These past studies showed that inhibitory ATL rTMS induced slowed RT during semantic processing. We note here that a recent study employing a shorten version of cTBS (20 s, 300 pulses) over the temporal pole showed a partial facilitatory effect on semantic processing (Bonni et al., 2015). cTBS over the right temporal pole was only found to improve RT during picture-based semantic processing in comparison to control stimulation (vertex) whilst there was no TMS effects after the left temporal pole stimulation in both picture and word semantic association tasks. These findings are inconsistent with the many previous ATL rTMS studies but it should be noted that (a) they delivered only half of the typical dose for the cTBS protocol (40 s, 600 pulses) (Huang et al., 2005), and (b) that inhibitory rTMS protocols may produce behavioural facilitation on higher cognitive domains due to the alerting effect of TMS (Vallar and Bolognini, 2011) or uncontrolled practice effects. In comparison, our study was able to establish and evaluate optimal protocols for inhibitory and excitatory rTMS for vATL-related semantic processing, and to demonstrate these effects over and above any alerting or practice.

Evidence from magnetoencephalography (MEG) and electroencephalography (EEG) have showed that semantic processing involves in multiple oscillatory brain activity. Specifically, semantic processing is associated with increases in synchronized activity in the theta (~5 Hz) and gamma (> 40 Hz) band and desynchronization in the alpha (8–12 Hz) and beta (13–35 Hz) (Doppelmayr et al., 2005; Hanslmayr and Staudigl, 2014; Rohm et al., 2001). Mollo et al. (Mollo et al., 2017) investigated brain oscillatory dynamics supporting semantic cognition with MEG and reported increased power in the theta and gamma and decreases in alpha and beta frequencies in the ATL. The desynchronization in the beta band potentially reflects functional inhibition, which allow more information to be maintained and processed in a cortical region by blocking interfering processing (Hanslmayr et al., 2012). Our 20 Hz rTMS protocol targeted the beta oscillatory activity in the ATL, which leads to semantic enhancement. It might be resultant from modulating the beta band such as an increase in power or phase alignment of the population activity to 20 Hz rTMS - entrainment of neural oscillation (Hanslmayr et al., 2019). In our category judgement task, to select the correct response (dog), it requires processing of semantic features in given stimuli (e.g., collie, dog, and car) and suppress irrelevant meaning (car) – inhibition. The entrainment driven from 20 Hz rTMS may enable the ATL to conduct this processing more efficiently resulting in semantic enhancement. To test it, future studies will be needed combining TMS with EEG or MEG.

The underlying mechanisms of rTMS effects are not fully understood but studies have showed that the most likely mechanisms relate to changes in synaptic transmission between neurons including long-term potentiation (LTP) and long-term depression (LTD) (Pell et al., 2011). Low frequency rTMS (~1 Hz and cTBS) can induce a suppression of cortical excitability whereas HF rTMS (5–20 Hz and iTBS) potentiate it (Fitzgerald et al., 2006). These effects have been found to depend on both γ-aminobutyric acid (GABA) and glutamate system (Funke and Benali, 2011; Lenz et al., 2016). Recent animal studies have unveiled the underlying cellular and molecular mechanisms of rTMS in vivo and in vitro (Muller-Dahlhaus and Vlachos, 2013). HF rTMS leads to long-lasting structural and functional changes in excitatory and inhibitory post-synapses. For example, 10 Hz rTMS (9 trains of 100 pulses with an interval 30 s) applied over the slice cultures of rat CA1 pyramidal neurons, increased excitatory post synaptic transmission, dendritic spin size in excitatory synapses (Vlachos et al., 2012) and decreased inhibitory synaptic transmission accompanied with the reduction in related inhibitory receptor properties (GABA) (Lenz et al., 2016). Our investigation of the neurochemical mechanism of the ATL function demonstrated that GABAergic action in the ATL plays a crucial role in semantic processing (Jung et al., 2017). Therefore, the observed facilitatory rTMS effects in the current study may be attributed to these molecular mechanisms underlying synaptic plasticity.

Accumulating evidence indicates that rTMS causes not only local changes, but also modulation of remote but functionally connected brain regions (Andoh and Paus, 2011; Bestmann et al., 2008; Esslinger et al., 2014; O'Shea et al., 2007; Sack et al., 2005). Thus, the long-term after effects can be attributed to activity changes in a given network rather than a local excitation or inhibition of an individual region alone. Our previous studies employing rTMS combined with fMRI showed that inhibitory rTMS over the left vATL reduced activity in the target site as well as upregulation in the contralateral vATL (Binney and Ralph, 2015; Jung and Lambon Ralph, 2016) and increased inter-ATL functional connectivity. Another study employing the same approach demonstrated that the cTBS over the left inferior frontal gyrus (IFG) induced increased activation in the homologues right IFG and enhanced the IFG-connectivity associated with better speech production (Hartwigsen et al., 2013). Similarly, the enhanced semantic performance after 20 Hz rTMS over the left ATL might be attributed to not only the increased cortical excitability at the target site but also the enhanced connectivity within the semantic network. Our recent investigation examining the semantic network showed that there were up-regulated regional activation in the bilateral ATL, left IFG and posterior middle temporal gyrus (pMTG) and the increased functional connectivity between the semantic regions (e.g., the ATL-connectivity, ATL-IFG connectivity, and IFG-pMTG connectivity) in the challenging semantic decision making (Jung and Lambon Ralph, 2019). The enhanced connectivity within the semantic network contributed to better semantic performance with increased task demands. In addition, studies with post-stroke aphasia patients combining rTMS with fMRI showed the evidence for the interhemispheric connectivity changes within the language network following the rTMS (Griffis et al., 2016; Hartwigsen et al., 2020; Szaflarski et al., 2011). These studies suggest that rTMS targeting a cortical area induces a large-scale network changes related to task processing leading to the short-term neuroplasticity of cognitive function. Therefore, the semantic benefits from 20 Hz rTMS over the ATL may be achieved by alterations in functional connectivity of the semantic network. Future studies that combine excitatory 20 Hz vATL rTMS with fMRI will be able to explore whether its positive effects reflect changes in the stimulated region and/or network-level modulations.

While our study is experimental in nature, these results are relevant to the development of potential therapeutic approaches in patients with semantic impairment (e.g., dementia and stroke) (Jefferies and Lambon Ralph, 2006; Lambon Ralph, 2014; Lambon Ralph et al., 2001). rTMS has repeatedly demonstrated facilitatory effects in rehabilitation after stroke (Bashir et al., 2010; Coslett, 2016; Griffis et al., 2016; Wang et al., 2012). Similar use of rTMS has also been suggested in improve memory and language function in patients with Alzheimer's disorder as well as the elderly healthy people (Cotelli et al., 2011, 2006, 2008; Sole-Padulles et al., 2006). These studies provide strong evidence for the potential clinical usefulness of rTMS. Here, our results provides insights into rTMS therapy and improvement in semantic function employing 20 Hz rTMS over the ATL.

It is generally agreed that the effect of rTMS is primarily determined by the specific combination of stimulation frequency and intensity (Wassermann et al., 2008). However, many other factors can influence rTMS effects such as history of synaptic activity, attention, time of day, and age (Ridding and Ziemann, 2010). In this study, we tried to control these nuisance factors in order to delineate task-specific facilitatory TMS effects on higher cognition. The pilot study illustrated that there were huge practice effects (the baseline of 5 sessions showed gradual reduction in RT according to the order of the session) and, even in the sham stimulation, practice effects were prominent. Thus, we included a substantial task familiarization procedure to minimize practice effects and to stabilize task performance prior to the main experiment, and each rTMS protocol was conducted at least a week apart. As a result, non-specific improvements in task performance were saturated prior to the TMS sessions (Fig. 2A) allowing us to be much more sensitive to TMS-induced changes in semantic performance.

There are several limitations in this study. First, the sham stimulation was always prior to the active rTMS. In order to control for the order effect, we conducted a mixed model analysis with TMS, task, and session as the main factors as well as the order (sham: 1, active rTMS: 2) as a covariate. This mixed model analysis confirmed our original findings revealing a significant main effect of TMS (F2, 14,401=28.53, *p* < 0.001), task (F1, 14,401=5.92, *p* = 0.015), and session (F2, 14,401 = 28.04, *p* < 0.001). There were significant 2-way interactions in TMS × task (F2, 14,401 = 9.40, *p* < 0.001), TMS × session (F2, 14,401 = 11.42, *p* < 0.001), and task × session (F1, 14,401 = 3.91, *p* = 0.048) as well as a 3-way interaction of TMS × task × session (F2, 14,401=16.78, *p* < 0.001). These results indicate that the order of sham stimulation did not affect our findings. Second, we performed the planned paired t-tests for our main comparisons of interest (e.g., comparisons between pre and post sessions and between 1 Hz, 20 Hz, and sham post sessions). In order to ensure our results, we performed post-hoc analysis with a multiple comparison correction from the mixed model analysis. Post-hoc paired t-tests with the Bonferoni correction (*p* < 0.0042) confirmed our findings, demonstrating RT for the category judgements was slower after the 1 Hz stimulation (*t* = −3.13, *p* = 0.002) and faster after the 20 Hz stimulation (*t* = 6.91, *p* < 0.001). The facilitatory effect after 20 Hz stimulation was significant compared to 1 Hz (*t* = 7.72, *p* < 0.001) and sham stimulation (*t* = 9.99, *p* < 0.001). Third, we unavoidably used different intensities for the inhibitory and facilitatory rTMS protocols. The intensity of TMS is one factor known to influence TMS effects such that stronger intensities produce bigger TMS effects. Previous studies have demonstrated that 1 Hz stimulation with RMT lower than 120% did not produce inhibitory effects in the motor cortex (Baudewig et al., 2001; Bohning et al., 2000; Jung et al., 2020). In terms of the excitatory stimulation, the safety guidelines recommend not to use intensities greater than 80% RMT for 20 Hz rTMS (Rossi et al., 2009). Thus, we employed 120% RMT for 1 Hz stimulation and 80% RMT for 20 Hz stimulation to ensure the expected TMS effects.

In conclusion, we identified an optimal vATL rTMS protocol, which can induce either semantic enhancement or inhibition in healthy individuals. Our results demonstrated that 20 Hz rTMS over the left vATL induced semantic performance enhancement – faster RT during a category judgment task, contrasting with 1 Hz rTMS – slower semantic decisions. Our findings not only add important new causal evidence for the vATL as the major hub for semantic representation but also indicate that HF rTMS could be a potential therapeutic tool in cognitive rehabilitation for patients with semantic impairments.

## Data code

The data that support the findings of this study are available on request from the corresponding author, J.J and M.A.L.

## Author contributions

J.J and M.A.L conceptualized and designed the study. J.J collected and analysed the data. J.J and M,A.L wrote the paper.

## Declaration of Competing Interest

The authors declare no competing financial interests.

## Appendix Supplementary materials

Image, application 1
